# The Role of Wearable Devices in Multiple Sclerosis

**DOI:** 10.1155/2018/7627643

**Published:** 2018-10-10

**Authors:** Maddalena Sparaco, Luigi Lavorgna, Renata Conforti, Gioacchino Tedeschi, Simona Bonavita

**Affiliations:** ^1^1st Clinic of Neurology, University of Campania “Luigi Vanvitelli”, Piazza Miraglia, 2, 80138 Naples, Italy; ^2^Neuroradiology Service, Department of Radiology, University of Campania “Luigi Vanvitelli”, C/o CTO Viale dei Colli Aminei 21, Naples, Italy; ^3^MRI Research Center SUN-FISM, University of Campania “Luigi Vanvitelli”, Naples, Italy; ^4^Institute for Diagnosis and Care “Hermitage Capodimonte”, Naples, Italy

## Abstract

Multiple sclerosis (MS) is the most common neurological disorder in young adults. The prevalence of walking impairment in people with MS (pwMS) is estimated between 41% and 75%. To evaluate the walking capacity in pwMS, the patient reported outcomes (PROs) and performance-based tests (i.e., the 2-minute walk test, the 6-minute walk test, the Timed 25-Foot Walk Test, the Timed Up and Go Test, and the Six Spot Step Test) could be used. However, some studies point out that the results of both performance-based tests and objective measures (i.e., by accelerometer) could not reflect patient reports of walking performance and impact of MS on daily life. This review analyses different motion sensors embedded in smartphones and motion wearable device (MWD) that can be useful to measure free-living walking behavior, to evaluate falls, fatigue, sedentary lifestyle, exercise, and quality of sleep in everyday life of pwMS. Caveats and limitations of MWD such as variable accuracy, user adherence, power consumption and recharging, noise susceptibility, and data management are discussed as well.

## 1. Introduction

Multiple sclerosis (MS) is a multifactorial demyelinating disease of the central nervous system and it is the most common neurological disorder in young adults, usually occurring between ages 20 and 40, especially in women [1, 2]. It is characterized by a large spectrum of symptoms and signs, involving several functional systems (pyramidal, cerebellar, sensory, brainstem, bowel and bladder, visual, mental, and ambulation) [3]. In particular, spasticity, fatigue, muscle weakness, balance problems, and abnormal walking mechanics are responsible for motor dysfunctions that may involve both upper and lower limbs; as a consequence functional independence and quality of life of people with MS (pwMS) are inevitably affected [4–6].

The prevalence of walking impairment in MS is estimated between 41% [7] and 75% [8]. The evaluation of ambulation has a prominent role in scoring MS-related disability with the Expanded Disability Status Scale (EDSS) [3], the Patient Determined Disease Steps (PDDS) [9, 10], and with the Timed 25-Foot Walk Test (T25FW) included in the MS Functional Composite [11]. Moreover, in pwMS several tests have been developed to evaluate walking endurance, such as the 2-minute walk test [12], the 6-minute walk test (6MW) [13], stability such as the Timed Up and Go Test [14], and the Six Spot Step Test [15]. However, walking tests have some drawbacks, indeed to evaluate walking distance, velocity and endurance, an adequate physical space and trained neurological staff are required [16].

Another important limit of these tests is that they do not represent patient performance in the real world. To evaluate the walking capacity in daily life, patient reported outcomes (PROs; i.e., 12-Item Multiple Sclerosis Walking Scale, MSWS-12 [17]) could be used; however, the correlation among PROs, walking tests, and objective performance measures is a strongly debated theme. Indeed, results of both performance-based tests [18] and objective measures (i.e., by accelerometer) [19] could not reflect PROs on walking performance. As a consequence, objective measures acquired everyday are needed to monitor the impact of MS on patient's daily life.

In this review we aim to examine different motion wearable devices (MWD), to evaluate their use in pwMS, and to highlight their advantages and limits.

## 2. Motion Wearable Devices (MWD)

In the last years, the large development of “health technologies" [20, 21] has enabled their use in patients' daily life for many reasons: ease to use, reliability, wide availability, nonintrusive monitoring, and support by different operating systems.

Nowadays, MWD offer the possibility to evaluate several parameters: range of movement, meters or number of steps in a day, walking speed, burnt calories, heart rate, and sleeping hours and also to have a feedback on physical activity (PA) [22–24]. These devices give the opportunity of continuous monitoring at home [25–29] for a long time (weeks, months, or years); therefore they can be used to measure objective outcomes [26–28]. Smartphones and MWD can also automatically record user interactions and collect continuous high-density data. Their internet connectivity and ability to store data can improve efficiency over other intermittent and limited methods [26], such as questionnaires and traditional pedometers. Particularly, different types of motion sensors, like pedometers, accelerometers, gyroscopes, inclinometers, grip sensors, and multisensors, are embedded in smartphones and MWD [30].

The pedometer is useful for measuring the number of steps per day; thus mechanical pedometers are called “step counters." They are the simplest wearable sensors to measure human motion, detecting the impact produced by steps using a spring-loaded mass or other switch mechanism. However, pedometers cannot record movement intensity, resulting in inaccurate evaluation of energy expenditure [31], although new pedometers could overcome this problem. More recently, a popular method of PA self-monitoring is represented by the use of accelerometers. They are sensors which measure the acceleration (change of speed in a time span) of objects in motion along reference axes (single-, dual-, and triaxis sensors). Acceleration can record intensity and frequency of human movement. The measures can be used to get information about speed and displacement, integrating accelerometer data with time. The common principle of accelerometers is based on a mechanical sensing element, composed by a proof mass (or seismic mass) connected to a mechanical suspension system compared to a reference frame. The proof mass deflects for inertial force due to acceleration or gravity, according to Newton's Second Law; therefore the displacement of proof mass can be used to measure acceleration [32, 33].

Historically, accelerometer output has been accepted as a measure of PA in healthy people [34, 35] and more recently this device has been applied to evaluate also people with a disease. Particularly, accelerometers may provide objective measures of real-life walking of people with neurological diseases [36].

In MS, a recent review [37], based on 32 articles, reports that uniaxial accelerometers are the most popular tools (68%) for objective measurement of PA. Pedometers (14%) and multisensor systems (3%) are the second and third ones. The most commonly acquired measures are activity counts per day (*n* = 21 studies) and steps per day (*n* = 11 studies).

Gait kinematics impairment, even in early stages of MS, would affect the normal sinusoidal vertical displacement of body's center during ambulation, which is the signal detected by the vertical-axis of accelerometer. This underlines that change in walking mobility may not necessarily affect PA [38].

Measures acquired by an accelerometer worn by pwMS for a 7-day period are correlated to scores at the MSWS-12, 6MW and T25FW [38–40], EDSS, and PDDS [41], to the oxygen cost of walking [42] and to gait parameters [43] such as speed, cadence, step time, step length, and time spent in double or single support [41]. For shorter periods, measures obtained by accelerometer worn for a 4-7 days period are correlated to EDSS and Rivermead Mobility Index scores [44, 45].

The number of steps/day provides a reliable and valid measure of daily life walking behavior in MS [46] as demonstrated by a test-retest reliability over a two-week time span [47].

One-point change in PDDS and 10-point change in MSWS-12 can be detected in a clinically meaningful change of 779 steps per day (14% of mean score for MS sample) [48].

Demographic and clinical features of pwMS taking fewer steps/day with motion devices correspond to being male, unemployed, with <13 years of education, progressive MS, higher levels of disability, and with longer disease duration [49].

MWD may be also used to investigate falls occurrence in pwMS. Fear of falling may induce a decrease in PA; indeed approximately 64% of pwMS worry of falling and, among those individuals, 83% report PA reduction [50].

By using an accelerometer, it has been shown that in a year pwMS, with self-reported falls, take significantly fewer steps than those who do not refer of falling (3510 versus 4940 steps/day;* P* <0.05). However, if controlled for disability, there is no significant difference between those who fall and those who do not (4092 versus 4373 steps/day;* P* >0.05) [51].

However, quality of data acquired by accelerometers should be tested because of possible gravitational 'cross-talk' when the wearable device is tilted, as it happens with falls. Improvement of accelerometer data quality may be reached by an adaptive filter applied before measuring dynamic pelvic sway patterns. Moreover, use of gyroscopic corrections and scaling filter thresholds by step frequency can improve wearable device accuracy (normalized root mean square error: ≤4.4%). PwMS present significantly greater pelvis sway range to compensate their lower limbs weakness and joint contractures; therefore visualization of asymmetric pelvic sway in pwMS allows for better understanding of their mobility impairment and planning of rehabilitation programs to reduce fall risk [52].

MWD can also measure inactivity time that may be detected by* posture sensors* characterized by an accelerometer in conjunction with gravitational components or through the alignment of the area of the body surrounding the pelvic area (i.e., pelvic alignment is different depending on standing, sitting, or lying). Another way to measure inactivity time is through* pressure sensors*, located in a sock, shoe, or chair. When the sensors are placed in a sock or shoe, the standing position increases pressure while the sitting position gives a reduction of pressure on the sensors. When the sensors are located on a chair, there is the opposite mechanism: when the users are sitting, the pressure sensor is active and when they stand up, the sensor is inactive [53]. Sensors application is very useful in pwMS; indeed they are less active than healthy people, and they have a sedentary lifestyle. Frau et al. described a reduction of PA reported by pwMS after the diagnosis (38% of participants stopped PA), especially for disease-related reasons [54].

Limited PAs may be detrimental to disability progression, mobility, quality of life, gait performances, stability, and muscle strength [55, 56]. Instead, exercise therapy has proven to improve motor impairment [57] and has positive influence on symptoms management through beneficial effects on fatigue, spasticity, mobility, depression, and pain [58]. Indeed, pwMS refer benefits from PA after diagnosis, preferring individual exercises rather than group activities [54].

A Cochrane review on the effect of exercise therapy (endurance training, muscle power training, task-oriented training, mixed training, or “other,” e.g., yoga) on MS related fatigue describes positive significant effects in favor of endurance training, mixed training, or “other” exercise therapy (e.g., yoga) compared to no PA. However, it has been suggested that the effects of exercise therapy on fatigue may be different among persons and may depend on the type of stimulus exercise [59].

Based on this evidence, telerehabilitation interventions are developing. A smartphone application (MS TeleCoach) for pwMS allows telemonitoring (through the device's integrated accelerometers and daily PROs administration) and telecoaching (with motivational messages, advice, and setting goal with increasing PA) and aims to reinforce self-management, to enhance PA levels, and to improve fatigue in pwMS [60].

Therefore, by acquiring data with new technological devices, it becomes possible to define an individual training, considering number of steps in a day, walking speed, and other specific parameters.

Digital health interventions (DHI) may be used to promote healthy behaviors and improve outcomes in people with a chronic disease [61]; in MS DHI seem to be beneficial in promoting PA [62–64].

Moreover, in pwMS, as well as in the general population, inactivity increases the risk of comorbidities (e.g., hypercholesterolemia, hypertension, obesity, and type 2 diabetes) that may have a detrimental effect on disability progression [65].

In the general population, MWD can be used to reduce sedentary lifestyle [53] through several modalities: furnishing a stimulus to reach a goal, a warning, or a vibratory feedback when the person does not move for a long time, incentives, gamification, or through social networks to promote competition among peers. In particular, these devices have goal-setting capabilities and customization of type and timing of feedback, based on age, sex, habits, and life-style, and they can be modified by user, if necessary; moreover, some devices have a sort of diary of progression toward a user-defined goal. In this way, people are stimulated to move; indeed, individuals who use pedometers increase their PA by 26.9% from baseline activity levels [66]. PwMS are quite apt to use technologies, especially if applied to health. A survey indicates that 93% of pwMS use the Internet compared to 75% of the general population [67] and nearly 90% of pwMS are interested in online information about maintaining a healthy lifestyle [68]. In a recent project, 248 participants enrolled from the online platform PatientsLikeMe [69] wore a device, for a mean of 18.2 days over a 21-day study period (adherence of 87%) and walked a mean of 4671 steps per day. At the end of the study, 191 participants responded to the poststudy survey: 88 % affirmed that the device was easy to use and to integrate into their daily routines; 83% reported interest in continuing to use the device after the study; and 68% believed that the device would have been useful for self-managing MS. So there is a possibility to integrate these technologies with everyday life of pwMS in order to measure and to increase PA, to improve everyday lifestyle, and to help training in rehabilitation.

MWD have also other biosensors, with different aims such as vital signs monitoring, especially heart rate monitoring, or sleep-devices, for characterizing duration and patterns of disruptive or restful sleep. Although insomnia occurs in over 40% of pwMS, compared to 10%–15% in the general population, only few studies investigated the use of sleep devices to study quality and quantity of sleep in pwMS highlighting the correlation between some features of sleep and fatigue, PA, and with the effect of some disease modifying therapies [70–73].

To our knowledge, MWD to evaluate tremor in pwMS have not been used yet. However, few studies had shown that these technologies could be used to evaluate essential tremor (ET) [74, 75] and tremor in Parkinson's disease [76]. In particular, a moderate-good correlation between MWD output (worn in the most tremulous wrist) and clinical tremor scores (at the Fahn-Tolosa-Marin tremor rating scale) was found in people with ET [74]. In the same group of patients, during the finger-to-nose maneuver (for kinetic tremor detection) no correlation was found between MWD output and clinical scores. These discrepancies, according to the different modalities of MWD application, may be due to different strategies chosen by patients with ET to complete the finger to nose task (different velocities of movements, different range of motion, etc.). In this perspective, the evaluation of kinetic tremor, the most frequent in pwMS, may be not thoroughly reliable with these devices.

An emerging alternative to wearable devices may be represented by non-wearable sensors, defined Ambient Measurement Systems (AMSs). AMSs are devices, placed on a top shelf in the patient's house, which measure and interpret human movements using a touchless sensor that acquires simultaneous information on patient's activity and on the surrounding context (environment), during normal daily activities. In MS the application of AMSs, although promising, has still several limitations thus requiring further technical development [16].

## 3. Limitations

Although these digital devices proved to be useful in the evaluation and management of pwMS they still have several limitations, including (a) variable accuracy depending on the type of sensors, the location on body surface, and abnormal movements during ambulation for disability; (b) user adherence (e.g., by forgetfulness or unwillingness); (c) power consumption and recharging; (d) noise susceptibility (e.g., due to placement or environment); and (e) data management [77].

Previous research [78] has indicated decreased accuracy of sensors in pwMS with higher levels of disability. In particular, accelerometers have high accuracy to measure steps taken under comfortable walking speed and faster walking speed conditions, but variable accuracy under the slower walking speed condition, particularly in pwMS with severe disability (e.g., ataxic or spastic gait). Therefore, new sensors in people with mild walking impairment should be tested for accuracy before expanding the application to people with more severe disability.

Accuracy and precision of different smartphone applications and MWD for measuring steps when walking on a treadmill, were evaluated considering also the body area where the device was worn (waist-worn, wrist-worn, and smartphone applications). The results suggest that devices based on waist-worn triaxial accelerometer are the most precise and accurate sensors [79, 80] because the waist is the barycenter of the whole human body. This implies that accelerations measured by a single sensor at this location can better represent the major human motion. Furthermore, waist-placement causes less constraint in body movement and discomfort can be minimized as well. According to the accelerations measured from a waist-worn accelerometer, it is possible to classify a range of basic daily activities, including walking, postures, and activity transitions [81–83].

User adherence is another important issue when using sensors to monitor walking in daily life; indeed possible mistaken characterization of true activity due to infrequent or sporadic usage may occur [84]. Furthermore, there is high variability in PA from day to day, as underlined by some studies that have shown that activity patterns can vary according to the day of the week and patient's demographic features [85]. Norris et al. [86], using an approach that examines reliability coefficients from random 2-day to 7-day averages, find that a minimum of any random 2-day observation is required to calculate a reliable mean daily step count in unaided walking. However, this study is conducted in a relatively small sample of pwMS with low disability. Similar findings are reported in a secondary analysis conducted in pwMS enrolled from the PatientLikeMe platform: averaging 2 days of step counts provides an adequate level of reliability (intraclass correlation coefficient, ICC, ≥0.7), which becomes a very high level of reliability (ICC 0.9) considering averaging data for a whole week [87].

To reduce mistaken characterization of true activity due to infrequent or sporadic usage it is possible to adopt several data management rules: excluding data above a threshold value of use of device (e.g., ≥3 days/week, ≥8 h/day), removing outlier data, and use averaging values for multiple days to improve reliability coefficients [85].

Furthermore, the increased performance under observation is another important effect of wearable devices considered both a positive factor, to motivate PA, or a negative one, because of the Hawthorne effect [88] that may influence people to modify their behavior in response to the awareness of being observed. In this perspective the acquired parameters may not define measure of true everyday activity.

A discussed issue on development and spread of wearable technologies is the reliability, privacy and security of data. These devices acquire and store several information: geographical location, living habits, heart rate, sleeping hours, everyday PA, account password and conversation, pictures, etc. Globally, the wearable technology regulation is in progress although there are several countries that have no regulatory framework, whereas other countries have an embryonic regulatory system [89, 90].

## 4. Discussion and Conclusion

This review reports the state of the art about the use of MWD in MS, explaining their role and reliability in detecting signs and symptoms. Considering the importance of motor impairment in reducing functional independence and in worsening quality of life of pwMS, MWD can be useful instruments to measure free-living walking behavior, to evaluate features of everyday life of pwMS, as falls, fatigue, sedentary lifestyle, exercise therapy, quality of sleep.

This review analyses also the propensity to use health-technologies by pwMS. These devices, though easy to use, reliable, widely available, and support different operating systems, have also several caveats and limitations: variable accuracy (e.g., by type of sensors, location on the body, and abnormal movements during ambulation due to disability); user adherence (e.g., by forgetfulness or unwillingness); power consumption and recharging; noise susceptibility (e.g., due to placement or environment) data management (e.g., reliability, privacy and security).

Future studies planned to overcome these caveats and limitations and the development of standardized protocols for the application in MS would be useful to improve and uniform patients' management through different MS centers.
